# Effects of high-intensity interval training on negative affect in adolescents with obesity: an exploratory analysis of the role of self-efficacy

**DOI:** 10.3389/fped.2026.1977400

**Published:** 2026-10-14

**Authors:** Shihao Wang, Chang Liu, Yali Xu

**Affiliations:** School of Physical Education, Henan University, Kaifeng, China

**Keywords:** adolescents with obesity, body composition, cardiorespiratory fitness, high-intensity interval training, negative affect, randomized controlled trial, self-efficacy

## Abstract

**Objective:**

To investigate the effects of an 8-week school-based high-intensity interval training (HIIT) program on body composition, cardiorespiratory fitness, negative affect, and self-efficacy in adolescents with obesity, and to explore whether self-efficacy statistically mediates the association between HIIT and negative affect.

**Methods:**

In this two-arm, parallel-group, randomized controlled trial, 55 adolescents with obesity (age 13.55 ± 1.21 years; BMI 29.58 ± 3.54 kg/m^2^) were randomly assigned to an experimental group (HIIT, *n* = 27) or a control group (regular physical education, *n* = 28). Body composition, estimated maximal oxygen uptake (VO₂max), negative affect (PANAS), and self-efficacy (GSES) were assessed before and after the 8-week intervention. The primary analyses were analyses of covariance (ANCOVA) with the post-intervention value as the dependent variable, group as the fixed factor, and the corresponding baseline value as the covariate; two-way repeated-measures ANOVAs (group×time) served as sensitivity analyses. An exploratory mediation analysis with 5,000 bias-corrected bootstrap resamples adjusted for baseline mediator, baseline outcome, sex, and age.

**Results:**

After adjustment for baseline values, the experimental group showed significantly lower post-intervention negative affect than the control group (adjusted difference −3.72, 95% CI [−4.81, −2.63], *p* < 0.001, partial *η*^2^ = 0.475) and significantly higher self-efficacy (adjusted difference 1.06, 95% CI [0.60, 1.53], *p* < 0.001, partial *η*^2^ = 0.290). Body weight, BMI, visceral fat mass, subcutaneous fat mass, body fat percentage, and fat mass were also significantly lower in the experimental group (all *p* < 0.001), and estimated VO₂max was higher (adjusted difference 1.54 mL/kg/min, 95% CI [0.86, 2.22], *p* < 0.001); the latter effect was attenuated but remained significant when post-intervention estimated VO₂max was recomputed with baseline BMI held constant (0.50, 95% CI [0.03, 0.98], *p* = 0.040), and was corroborated by the BMI-free 20-m shuttle-run lap count (*p* = 0.004). The exploratory mediation analysis did not support an indirect effect through self-efficacy (a × b = 0.09, bootstrap 95% CI [−0.75, 0.82]).

**Conclusion:**

Eight weeks of school-based HIIT improved body composition and estimated cardiorespiratory fitness, reduced negative affect, and enhanced self-efficacy in adolescents with obesity. The indirect effect through self-efficacy was not statistically supported after baseline adjustment; because the mediator and the outcome were measured concurrently, temporal ordering cannot be established, and the mediating role of self-efficacy remains an open question for longitudinal research.

## 1 Introduction

Adolescent obesity is a major global public health issue. A joint investigation by the NCD Risk Factor Collaboration (NCD-RisC) and the World Health Organization (WHO) found that the global prevalence of obesity among children and adolescents increased more than fourfold from 1990 to 2022 (1). Obesity not only increases adolescents' risk of chronic diseases such as hypertension, type 2 diabetes, and dyslipidemia (2, 3), but, as a visible physical characteristic, also frequently exposes them to social discrimination, peer rejection, and negative evaluation, thereby giving rise to a series of psychological adjustment problems (4). Negative affect, which is particularly common among adolescents, refers to the unpleasant and tense subjective experiences that arise when an individual responds to environmental stimuli, including sadness, anger, tension, and fear (5). Studies have shown that adolescents who remain in a state of high negative affect for prolonged periods are more likely to seek comfort through coping strategies such as overeating and avoidance of physical activity, thereby aggravating weight problems (6–8). In turn, worsening obesity caused by weight gain is associated with higher negative affect scores, often reflecting emotions such as distress, anger, disgust, fear, and shame (9). Adolescents with obesity also show a significantly higher risk of negative affect than their normal-weight peers (10). Obesity and negative affect thus form a vicious cycle, and identifying effective ways to break this cycle and improve the emotional health of adolescents with obesity is of urgent practical importance.

In addition, self-efficacy is regarded as a key protective cognitive variable. Proposed by Bandura, self-efficacy refers to an individual's belief in his or her ability to organize and execute actions required to achieve specific goals (11). This belief is formed and developed primarily through four sources of information: mastery experiences accumulated through personal successes and failures; vicarious experiences gained by observing the success of others with similar abilities; verbal persuasion in the form of positive external feedback; and the individual's subjective interpretation of physiological arousal and emotional states. Research indicates that individuals with high self-efficacy demonstrate greater resilience, more positive coping strategies, and less emotional distress when facing difficulties (12, 52). For adolescents with obesity, repeated setbacks in weight management and exercise can readily lead to low self-efficacy, which may further amplify negative affect (13). Conversely, improving their self-efficacy may strengthen their motivation and confidence to cope with the pressure of weight loss and maintain healthy behaviors, making it an important psychological resource for improving emotional well-being (14).

Exercise intervention is widely recognized as an integrated means of improving physical and mental health. Regular physical activity not only improves body composition and metabolic indicators but also directly promotes positive emotions and alleviates anxiety and depression (15). Cross-sectional surveys of university students have shown that higher levels of physical exercise are associated with lower negative affect scores and higher self-efficacy scores (16–18). High-intensity interval training (HIIT), one of the most popular exercise modalities, consists of alternating short bouts of high-intensity exercise lasting from several seconds to several minutes with periods of active or passive recovery, thereby producing substantial physiological stimulation within a relatively short total duration (19). Studies have shown that HIIT is at least as effective as moderate-intensity continuous training (MICT) in improving body composition, cardiovascular health, and insulin sensitivity in adolescents with obesity, and may offer greater time efficiency (20, 21). HIIT can also improve negative affect and enhance self-efficacy (22–25). Nevertheless, existing research has clear limitations. Most studies of HIIT in adolescents with obesity have focused on physiological outcomes, and research on the mechanisms underlying emotional improvement remains scarce. Moreover, previous studies have generally examined the effect of HIIT on either negative affect or self-efficacy as a single variable, without investigating whether self-efficacy can serve as a mediator linking exercise to emotional improvement. This mechanistic pathway has not yet been verified in adolescents with obesity through a rigorous experimental design.

Against this background, the present study proposes an integrated research framework. Adolescents with obesity received an 8-week structured, school-based HIIT intervention in a randomized controlled trial to systematically examine: (1) the effect of HIIT on negative affect in adolescents with obesity (primary outcome); (2) the effect of HIIT on self-efficacy (primary outcome) and on body composition and cardiorespiratory fitness (secondary outcomes); and (3) on an exploratory basis, whether self-efficacy statistically mediates the association between HIIT and negative affect when baseline values are taken into account.

## 2 Participants and methods

### 2.1 Study design and ethical approval

This study was a two-arm, parallel-group, randomized controlled trial conducted in a school setting, and is reported in accordance with the CONSORT statement. The study protocol was approved by the Biomedical Research Ethics Sub-Committee of Henan University (approval No. HUSOM2024-133, approved on March 1, 2024), and was conducted in accordance with the Declaration of Helsinki. The trial was not prospectively registered (see Limitations). At the design stage, the study was conceived as a minimal-risk, school-based health-promotion intervention, and prospective trial registration was not considered mandatory for this type of study; we now recognize that prospective registration would have been best practice. The primary and secondary outcomes and the measurement procedures were specified in the ethics-approved protocol before data collection began; the ANCOVA-based primary analyses and the bootstrap mediation reanalysis were conducted during revision in response to the reviewers' requests. Written informed consent was obtained from the parents or legal guardians of all participants, and written assent was additionally obtained from each adolescent participant before enrollment.

### 2.2 Participants

From March 31 to May 31, 2025, adolescents with obesity were screened at Changzhi No. 1 Middle School in Tongxu County, Kaifeng City, China. Obesity was defined according to the Chinese national standard “Screening for Overweight and Obesity Among School-Age Children and Adolescents” (WS/T 586-2018). A total of 63 adolescents with obesity were identified. Of these, 55 were enrolled and randomized; the remaining 8 were not enrolled for the following reasons: declined to participate (*n* = 3), did not meet the inclusion criteria (*n* = 4), and other reasons (*n* = 1) (Figure 1).

**Figure 1:**
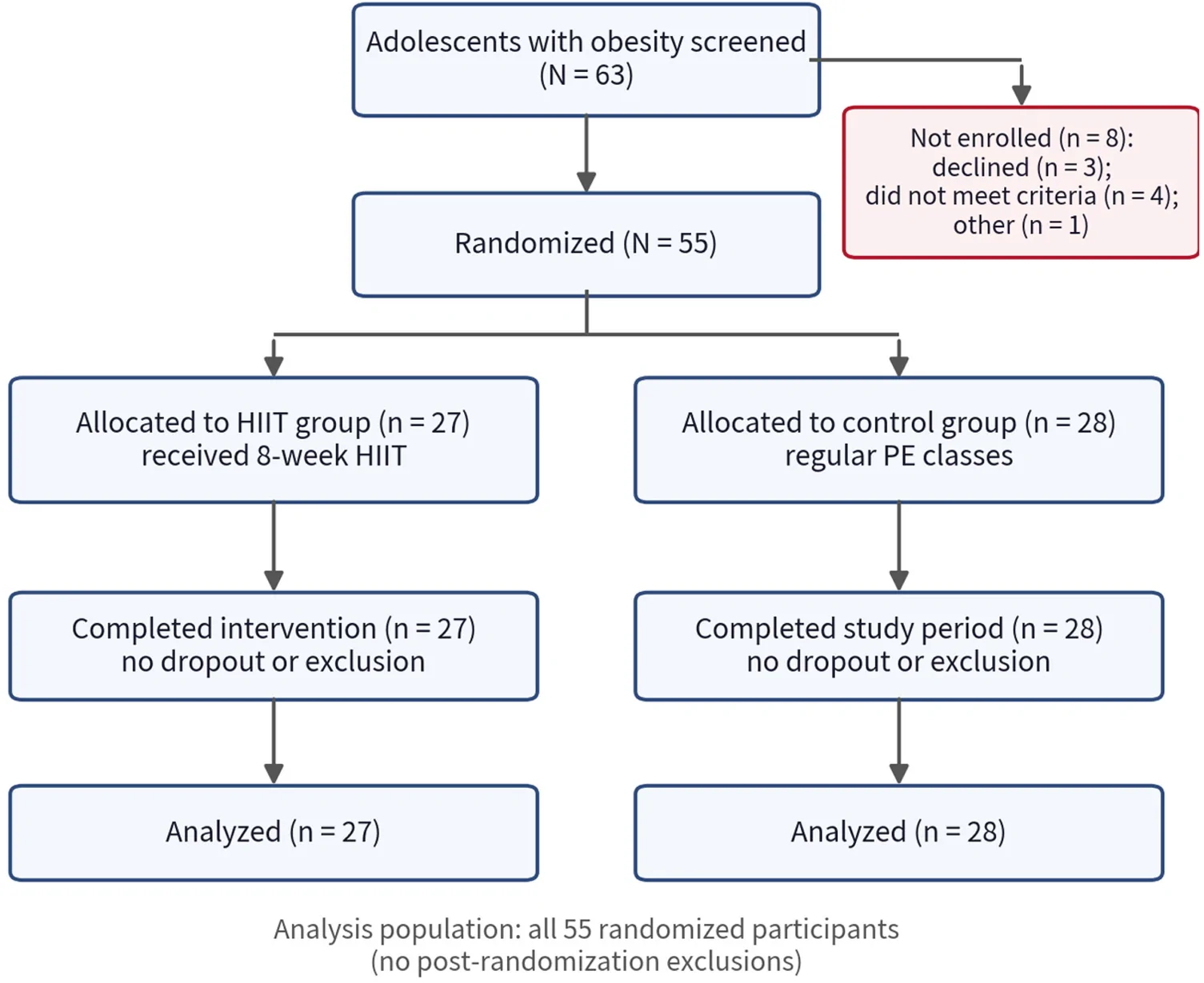
CONSORT-style participant flow diagram.

The inclusion criteria were: (1) age 12–15 years; (2) a BMI greater than or equal to the obesity cut-off for the corresponding sex and age group under WS/T 586-2018; (3) body-weight fluctuation of less than 2 kg during the previous three months; (4) no psychiatric or organic diseases; and (5) voluntary participation with parental consent and signed informed consent.

The exclusion criteria were: (1) cardiovascular or cerebrovascular disease; (2) severe mental illness; (3) surgery, trauma, or infection within the previous three months; and (4) orthopedic or neurological disorders limiting exercise capacity.

*A priori* dropout criteria were defined as follows: (1) consecutive absences totaling at least 20% of all training sessions (i.e., five or more absences among the 24 sessions delivered over 8 weeks); (2) a request by the participant or legal guardian to withdraw; (3) failure to complete any key pre- or post-intervention assessment; (4) independent participation in another systematic exercise, weight-loss, or psychological intervention program during the experiment; or (5) an exercise injury, illness, or health problem that, after evaluation, made continued training or testing inappropriate. No participant withdrew or was excluded after randomization: all 55 randomized participants completed the intervention and the post-intervention assessments and were included in the analysis (Figure 1). No *a priori* sample-size calculation was performed; the sample size was determined by the number of eligible adolescents with obesity at the participating school who consented to participate.

### 2.3 Randomization, allocation concealment, and blinding

Participants were randomly assigned to the experimental group or the control group using simple randomization. For each participant, a random number was generated using the RAND function in Microsoft Excel; participants were then ranked in ascending order of their random numbers and sequentially allocated to the experimental group (*n* = 27) and the control group (*n* = 28). The allocation sequence was generated and kept by a researcher who was not involved in participant recruitment, screening, or outcome assessment. Allocation concealment was implemented using sequentially numbered, sealed, opaque envelopes: after a participant had signed the informed consent form and been confirmed as eligible, the recruiting staff opened the next envelope in sequence to determine the group assignment. Given the nature of the exercise intervention, blinding of participants and intervention staff was not feasible; outcome assessors were blinded to group allocation.

### 2.4 Exercise intervention protocol

Because the participants had previously low levels of physical activity and their body size could make their exercise capacity lower than that of adolescents with normal weight, the training primarily used a running-based high-intensity, short-interval model. Each training session comprised three sets, with each set containing eight bouts. Each bout consisted of 15 s of high-intensity running followed by 15 s of low-intensity recovery running, with 3 min of rest between sets. During the high-intensity phase, exercise intensity was set at 100% of the individual's maximal aerobic speed (MAS); recovery intensity was set at 50% of MAS. Before the intervention, each participant's MAS was determined using a 20-m shuttle-run test, and the distance to be covered in 15 s was calculated from that individual's MAS. Training was conducted on the track and field ground of the participants' school three times per week (Monday, Wednesday, and Friday) for 8 weeks; each session lasted approximately 40 min, including an 18-minute core training component. All sessions were supervised and instructed on site by uniformly trained members of the research team (Figure 2).

**Figure 2:**
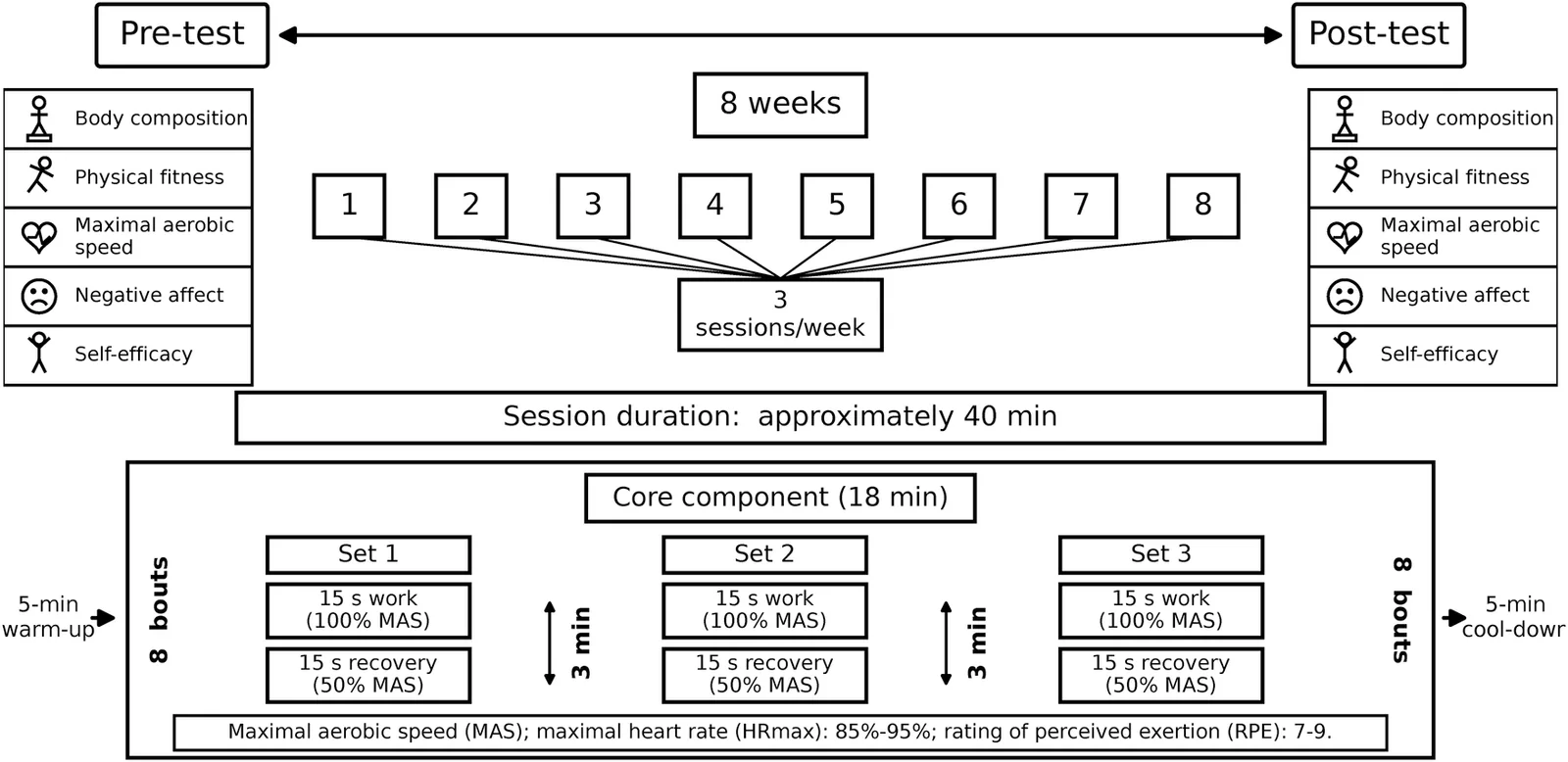
Experimental protocol. MAS, maximal aerobic speed; HRmax, maximal heart rate; RPE, rating of perceived exertion.

Intervention fidelity was monitored as follows. Attendance was recorded at every training session; the mean attendance rate was 97.7% of the 24 scheduled sessions. Exercise intensity was monitored using heart rate and the children's Rating of Perceived Exertion (RPE) scale: heart rate was estimated by counting the radial pulse for 6 s (multiplied by 10) immediately after each high-intensity bout, with a target of 85%–95% of the individual maximal heart rate, and RPE was recorded after each set, with a target score of 7–9 (from “very hard” to “extremely hard”). On the basis of these post-bout pulse checks and RPE ratings, the target intensity was judged to have been achieved in all sessions. Because pulse palpation after the 15-s bouts captures the early recovery heart rate rather than the peak in-bout intensity, this method may overestimate the actual exercise intensity (see Limitations). No exercise-related adverse events occurred during the intervention period.

### 2.5 Control condition

Participants in the control group attended their regular physical education classes (three sessions per week, mainly track and field, ball games, and basic physical fitness activities) and routine extracurricular physical activities, and received no structured training intervention. All participants were instructed to maintain their habitual diet and physical activity patterns and not to begin any new exercise, weight-loss, or psychological intervention program during the study period. Dietary intake and out-of-school physical activity were not objectively monitored (see Limitations).

### 2.6 Outcome measures

The primary outcomes were negative affect (PANAS negative affect subscale) and self-efficacy (GSES). Secondary outcomes were body-composition measures and estimated VO₂max.

Body composition. Body weight, body fat percentage, fat mass, fat-free mass, muscle mass, visceral fat mass, and subcutaneous fat mass were assessed using an InBody bioelectrical impedance analyzer under standardized conditions: participants wore light, close-fitting clothing, removed metal accessories, and stood barefoot holding the instrument's handles. All measurements were performed by the same trained research staff using the same device, at the same time of day for pre- and post-intervention assessments.

Maximal aerobic speed. A 20-m shuttle-run test was used; research has shown that this method effectively reflects aerobic capacity (26). The test was conducted on a field marked by two parallel lines 20 m apart. The initial speed was 8.5 km/h and increased by 0.5 km/h at each stage (approximately 1 min). A rhythmic audio recording (Beep Test) was played, and participants ran back and forth over 20 m in time with the audio. The test ended when a participant failed to reach the line before the audio cue on two consecutive occasions or voluntarily stopped because of exhaustion. The highest speed stage completed was recorded, converted to meters per second, and taken as MAS (Figure 3).

**Figure 3:**
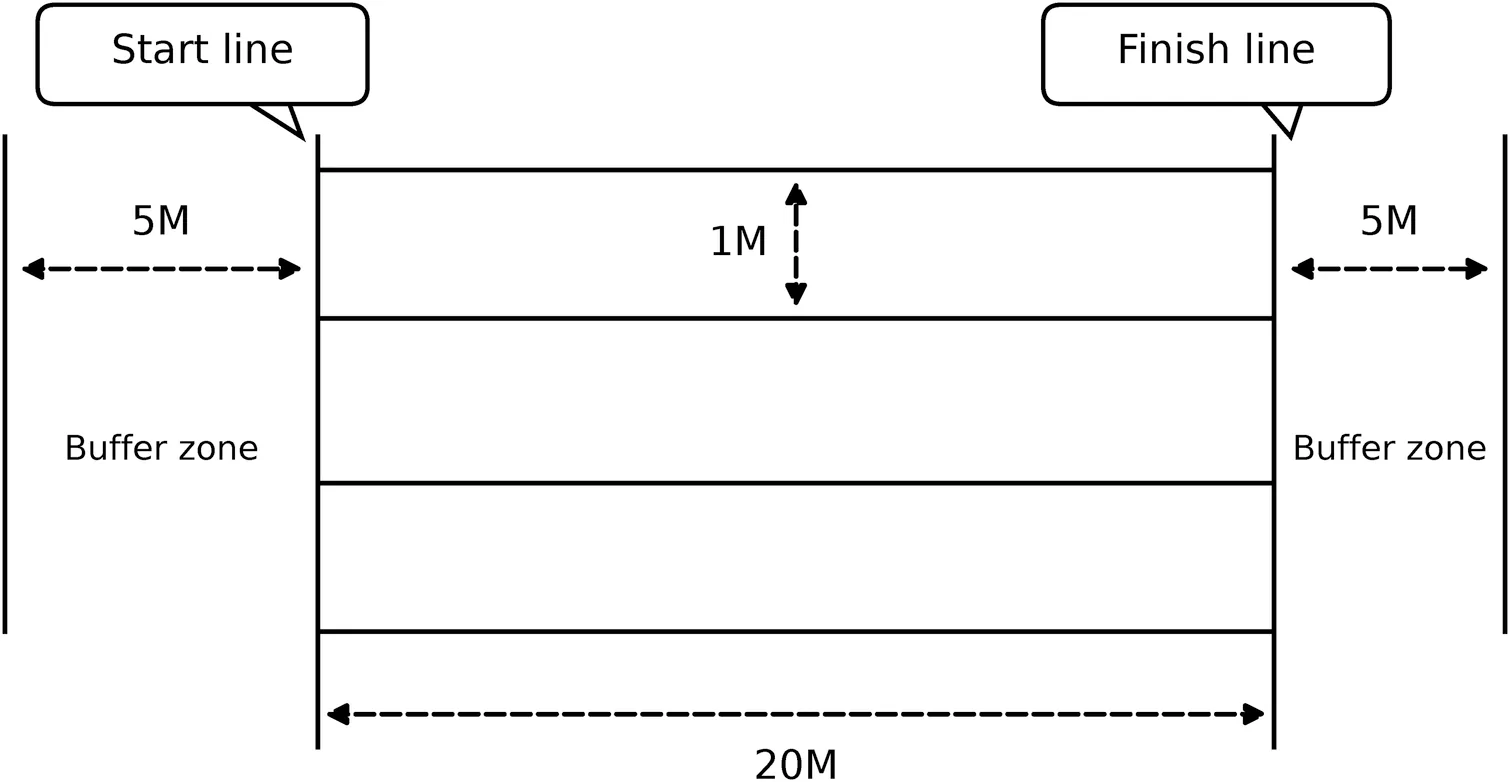
The 20-m shuttle-run test (20-m SRT).

Cardiorespiratory fitness. Maximal oxygen uptake (VO₂max) was estimated from the 20-m SRT using the equation (27): VO₂max = 62.103 + (0.302 × laps)−(0.877 × BMI)−(4.874 × sex)−(0.465 × age), where male = 0, female = 1, and laps denotes the number of completed 20-m SRT laps. Throughout this manuscript, VO₂max values are referred to as “estimated VO₂max” because they were derived from a prediction equation rather than measured directly. Because BMI is a term in this equation and BMI changed over the intervention, a sensitivity analysis was therefore performed (see Section 2.7).

Psychological outcomes. Negative affect was assessed with the negative affect subscale of the Chinese version of the Positive and Negative Affect Schedule (PANAS). The subscale contains 10 adjectives describing negative affect (e.g., “afraid,” “nervous,” and “irritable”) rated on a 5-point Likert scale (1 = not at all, 5 = extremely); participants rated the extent to which they had experienced these emotions during the previous two weeks. In the present study, the Cronbach's alpha coefficient of this subscale was 0.797. General self-efficacy was assessed with the Chinese version of the General Self-Efficacy Scale (GSES) revised by Wang Caikang et al. (28), which contains 10 items rated on a 4-point Likert scale (1 = not at all true, 4 = exactly true; total range 10–40, higher scores indicating higher self-efficacy); the Cronbach's alpha coefficient in the present study was 0.714.

### 2.7 Statistical analysis

Data are expressed as mean ± standard deviation. The significance level was set at two-sided *p* < 0.05. Normality was examined with the Shapiro–Wilk test and homogeneity of variance with Levene's test. The primary between-group analyses were analyses of covariance (ANCOVA) for each outcome, with the post-intervention value as the dependent variable, group as the fixed factor, and the corresponding baseline value as the covariate; the homogeneity-of-regression-slopes assumption was examined for each outcome before fitting the final models. Adjusted means, adjusted between-group differences with 95% confidence intervals, F statistics, and partial *η*^2^ (0.01 = small, 0.06 = medium, 0.14 = large) are reported. As a sensitivity analysis, two-way repeated-measures ANOVAs (group×time) were conducted. For outcomes showing departures from ANCOVA assumptions (body fat percentage, negative affect, and self-efficacy), results were verified with bootstrap ANCOVA using 5,000 resamples.

An exploratory mediation analysis examined whether self-efficacy statistically mediated the association between the intervention and negative affect, with group assignment as the independent variable (1 = HIIT, 0 = control), post-intervention self-efficacy as the mediator, and post-intervention negative affect as the outcome. Baseline self-efficacy, baseline negative affect, sex, and age were entered as covariates in all equations. The indirect effect (a × b) was tested with 5,000 bias-corrected bootstrap samples and was considered statistically supported when the bootstrap 95% confidence interval excluded zero (29, 30).

Because BMI is a term in the equation used to estimate VO₂max and BMI changed over the intervention, a sensitivity analysis recomputed post-intervention estimated VO₂max using each participant's baseline BMI; the number of 20-m shuttle-run laps completed, which does not involve BMI, was additionally analyzed as a BMI-free performance measure.

The analysis population comprised all 55 randomized participants (consistent with intention-to-treat principles, as no post-randomization exclusions occurred). ANCOVA, repeated-measures ANOVA, and descriptive analyses were performed with SPSS 26.0; the bootstrap procedures (bootstrap ANCOVA and the mediation analysis) were implemented in Python 3.11 (numpy/scipy, 5,000 resamples).

## 3 Results

### 3.1 Participant flow and baseline characteristics

Of the 63 adolescents with obesity screened, 55 were enrolled and randomized (experimental group, *n* = 27, 13 girls and 14 boys; control group, *n* = 28, 14 girls and 14 boys); all 55 completed the 8-week study and were included in the analysis (Figure 1). At baseline, the two groups did not differ significantly in demographic variables, body composition, estimated VO₂max, or psychological outcomes (all *p* > 0.05), indicating adequate baseline comparability between the two groups (Table 1).

**Table 1:** Baseline characteristics of the two groups (*N* = 55).

Outcome	Experimental group (*n* = 27)	Control group (*n* = 28)	p
Age (years)	13.48 ± 1.16	13.61 ± 1.29	0.705
Height (m)	1.61 ± 0.08	1.62 ± 0.09	0.673
Body weight (kg)	75.71 ± 12.61	80.08 ± 14.86	0.246
BMI (kg/m^2^)	28.96 ± 3.32	30.18 ± 3.70	0.202
Estimated VO₂max (mL/kg/min)	33.68 ± 4.78	31.68 ± 5.54	0.157
Visceral fat mass (kg)	3.13 ± 1.14	3.69 ± 1.58	0.140
Subcutaneous fat mass (kg)	20.08 ± 4.98	22.28 ± 5.86	0.140
Body fat percentage (%)	30.41 ± 4.51	32.08 ± 4.98	0.199
Fat-free mass (kg)	52.49 ± 8.20	54.10 ± 9.31	0.500
Total muscle mass (kg)	48.18 ± 7.54	49.50 ± 8.48	0.544
Fat mass (kg)	23.22 ± 6.02	25.98 ± 7.29	0.132
Negative affect	28.00 ± 4.45	28.43 ± 5.49	0.752
Self-efficacy	24.74 ± 2.88	22.96 ± 4.81	0.102

Data are mean ± SD. Between-group comparisons by independent-samples t tests.

### 3.2 Assumption checks

Shapiro–Wilk tests indicated that most outcomes were normally distributed within each group-by-time cell (*p* > 0.05); exceptions were body weight, fat-free mass, and total muscle mass (pre-intervention, experimental group), negative affect (pre-intervention, both groups; post-intervention, control group), and self-efficacy (pre-intervention, control group). Levene's test indicated homogeneity of variance for all outcomes except self-efficacy (pre-intervention *p* < 0.001; post-intervention *p* = 0.007). The homogeneity-of-regression-slopes assumption for ANCOVA was met for all outcomes (*p* > 0.10) except body fat percentage (*p* = 0.030). Because ANCOVA is robust to mild non-normality at this sample size, parametric ANCOVA was retained as the primary analysis, and the affected outcomes (body fat percentage, negative affect, and self-efficacy) were additionally verified with bootstrap ANCOVA (5,000 resamples); the bootstrap 95% confidence intervals [negative affect [−4.72, −2.65]; self-efficacy [0.63, 1.50]; body fat percentage [−2.57, −1.07]] led to identical conclusions.

### 3.3 Primary analyses: ANCOVA

After adjustment for baseline values, the experimental group showed significantly lower post-intervention body weight, BMI, visceral fat mass, subcutaneous fat mass, body fat percentage, and fat mass than the control group (all *p* < 0.001; partial *η*^2^ = 0.234–0.410, i.e., large effects), whereas fat-free mass and total muscle mass did not differ between groups (*p* = 0.576 and *p* = 0.183, respectively). Estimated VO₂max was significantly higher in the experimental group (adjusted difference 1.54 mL/kg/min, 95% CI [0.86, 2.22], *p* < 0.001, partial *η*^2^ = 0.282) (Table 2, Figure 4).

**Table 2:** Primary analyses (ANCOVA): baseline-adjusted post-intervention differences between the experimental and control groups (*N* = 55).

Outcome	Experimental, adjusted mean	Control, adjusted mean	Adjusted difference [95% CI]	F(1,52)	p	Partial *η*^2^
Body weight (kg)	75.97	78.29	−2.31 [−3.48, −1.15]	15.87	<0.001	0.234
BMI (kg/m^2^)	28.19	29.43	−1.24 [−1.70, −0.78]	28.89	<0.001	0.357
Visceral fat mass (kg)	3.09	3.53	−0.44 [−0.60, −0.27]	29.15	<0.001	0.359
Subcutaneous fat mass (kg)	19.97	21.60	−1.62 [−2.17, −1.08]	36.07	<0.001	0.410
Body fat percentage (%)	29.96	31.78	−1.82 [−2.57, −1.07]	23.86	<0.001	0.314
Fat-free mass (kg)	52.92	53.16	−0.24 [−1.10, 0.62]	0.32	0.576	0.006
Total muscle mass (kg)	49.02	48.41	0.62 [−0.30, 1.53]	1.82	0.183	0.034
Fat mass (kg)	23.07	25.12	−2.05 [−2.75, −1.36]	35.13	<0.001	0.403
Estimated VO₂max (mL/kg/min)	34.34	32.80	1.54 [0.86, 2.22]	20.46	<0.001	0.282
Negative affect	25.47	29.19	−3.72 [−4.81, −2.63]	47.12	<0.001	0.475
Self-efficacy	24.80	23.73	1.06 [0.60, 1.53]	21.21	<0.001	0.290

ANCOVA with the post-intervention value as the dependent variable, group as the fixed factor, and the corresponding baseline value as the covariate. Partial η^2^: 0.01 = small, 0.06 = medium, 0.14 = large.

**Figure 4:**
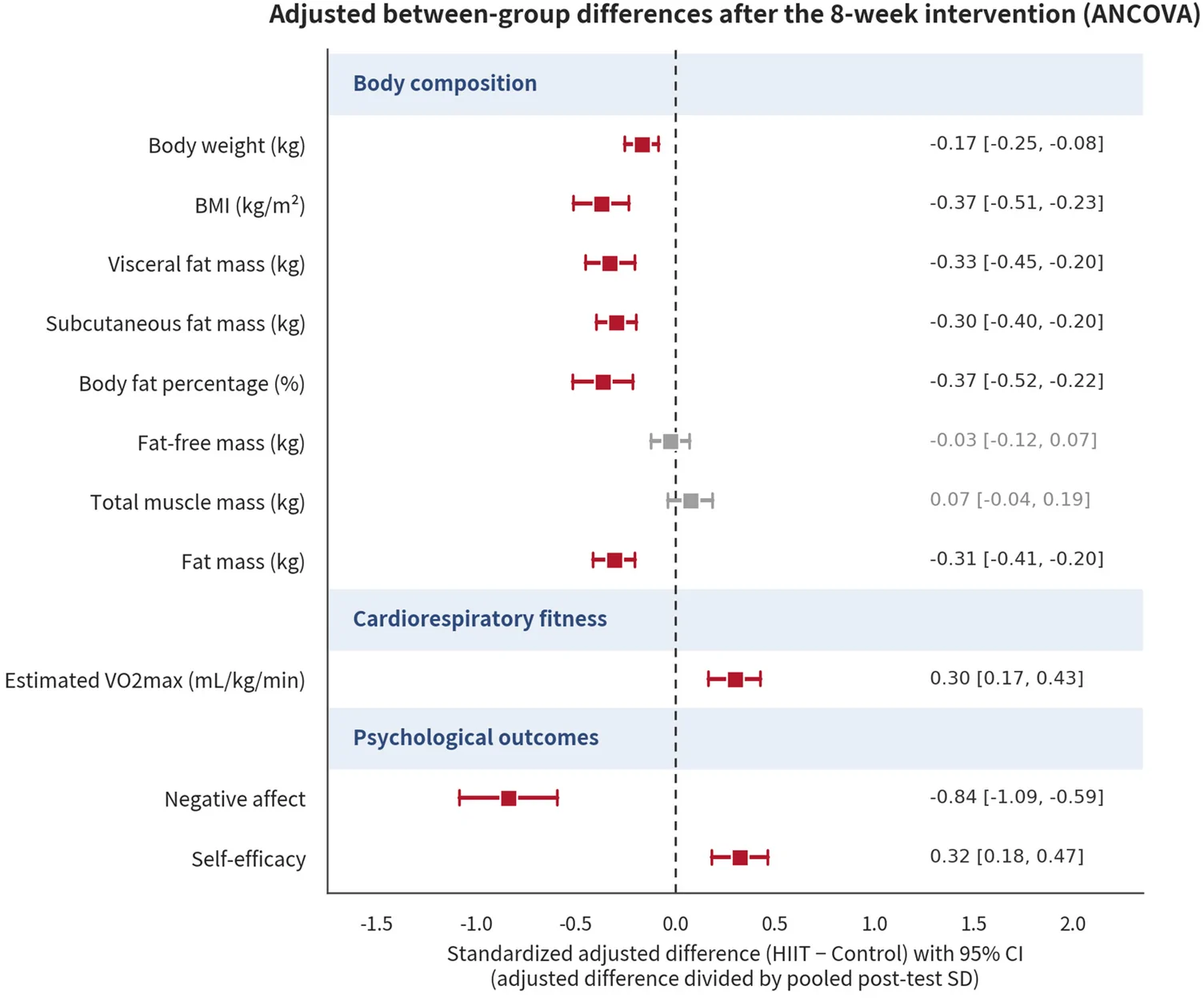
Standardized adjusted between-group differences (HIIT−control) with 95% confidence intervals after the 8-week intervention (ANCOVA). Estimates were standardized by dividing the adjusted difference and its confidence interval by the pooled post-intervention standard deviation.

For the primary psychological outcomes, post-intervention negative affect was significantly lower in the experimental group than in the control group (adjusted difference −3.72, 95% CI [−4.81, −2.63], F(1,52) = 47.12, *p* < 0.001, partial *η*^2^ = 0.475), and post-intervention self-efficacy was significantly higher (adjusted difference 1.06, 95% CI [0.60, 1.53], F(1,52) = 21.21, *p* < 0.001, partial *η*^2^ = 0.290) (Table 2, Figures 4, 5).

**Figure 5:**
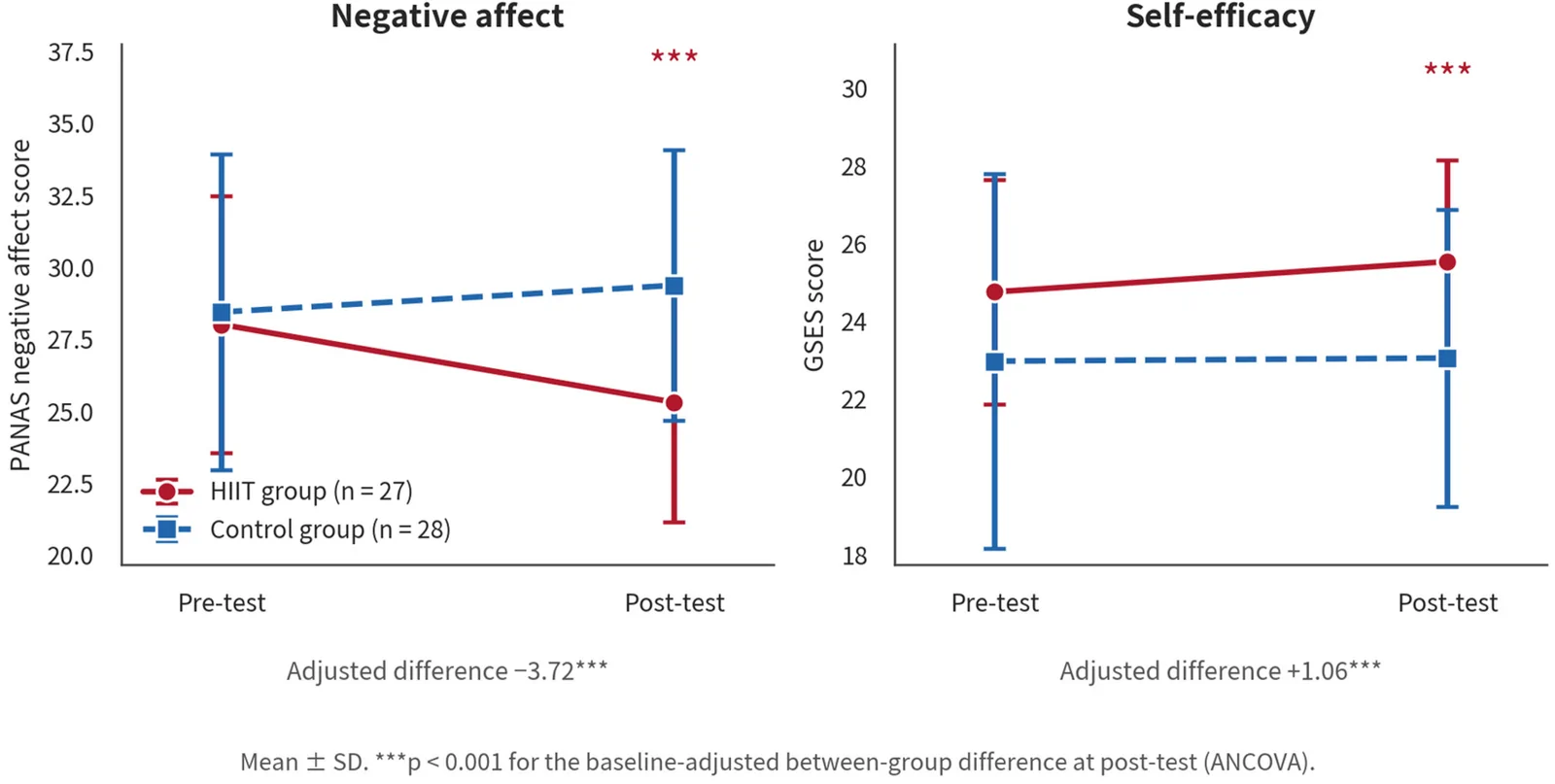
Negative affect (PANAS) and self-efficacy (GSES) scores before and after the intervention in the two groups (mea*n* ± SD).

### 3.4 Sensitivity analysis: group × time interactions

Two-way repeated-measures ANOVAs yielded conclusions identical to the primary ANCOVA: significant group × time interactions were observed for all outcomes (all *p* < 0.05) except fat-free mass and total muscle mass (Table 3).

**Table 3:** Sensitivity analysis: group × time interactions from two-way repeated-measures ANOVA (*N* = 55).

Outcome	Experimental *Δ* (post−pre)	Control *Δ* (post−pre)	F(1,53)	p	Partial η^2^
Body weight (kg)	−1.91	+0.32	15.23	<0.001	0.223
BMI (kg/m^2^)	−1.34	−0.20	22.63	<0.001	0.299
Visceral fat mass (kg)	−0.31	+0.09	23.64	<0.001	0.309
Subcutaneous fat mass (kg)	−1.21	+0.38	36.68	<0.001	0.409
Body fat percentage (%)	−1.30	+0.53	25.41	<0.001	0.324
Fat-free mass (kg)	−0.39	−0.15	0.32	0.574	0.006
Total muscle mass (kg)	+0.17	−0.44	1.80	0.185	0.033
Fat mass (kg)	−1.52	+0.47	34.70	<0.001	0.396
Estimated VO₂max (mL/kg/min)	+1.64	+0.16	19.93	<0.001	0.273
Negative affect	−2.70	+0.93	35.93	<0.001	0.404
Self-efficacy	+0.78	+0.07	5.15	0.027	0.089

*Δ* = within-group change from pre- to post-intervention. The interaction F statistic is equivalent to the squared independent-samples t statistic on the change scores.

### 3.5 Sensitivity analysis for estimated VO₂max

Because BMI is a term in the equation used to estimate VO₂max and BMI decreased in the experimental group, part of the observed between-group difference in estimated VO₂max could reflect a mathematical coupling with the change in BMI rather than a pure improvement in aerobic performance. When post-intervention estimated VO₂max was recomputed with each participant's baseline BMI held constant, the between-group difference was attenuated but remained statistically significant (adjusted difference 0.50 mL/kg/min, 95% CI [0.03, 0.98], *p* = 0.040, partial *η*^2^ = 0.079). The number of 20-m shuttle-run laps completed, which does not involve BMI, also improved significantly more in the experimental group (adjusted difference 2.12 laps, 95% CI [0.70, 3.54], *p* = 0.004, partial *η*^2^ = 0.147), corroborating a genuine improvement in running performance (Table 4).

**Table 4:** Sensitivity analyses for cardiorespiratory fitness (*N* = 55).

Measure	Adjusted difference [95% CI]	F(1,52)	p	Partial η^2^
Estimated VO₂max (post-intervention BMI in equation)	1.54 [0.86, 2.22]	20.46	<0.001	0.282
Estimated VO₂max (baseline BMI held constant)	0.50 [0.03, 0.98]	4.45	0.040	0.079
20-m shuttle-run laps completed (BMI-free)	2.12 [0.70, 3.54]	8.94	0.004	0.147

### 3.6 Exploratory mediation analysis

The exploratory mediation analysis examined whether self-efficacy statistically mediated the association between group assignment and post-intervention negative affect, adjusting for baseline self-efficacy, baseline negative affect, sex, and age (Table 5, Figure 6). The a path was significant: participants in the HIIT group had higher post-intervention self-efficacy (B = 1.06, *p* < 0.001). The b path was not significant: after adjustment for baseline values and covariates, post-intervention self-efficacy did not significantly predict post-intervention negative affect (B = 0.09, *p* = 0.811). The total effect (c = −3.67, *p* < 0.001) and the direct effect (c′ = −3.76, *p* < 0.001) were both significant. Critically, the indirect effect was small and not statistically supported [a × b = 0.09; bootstrap 95% CI [−0.75, 0.82] by percentile method; [−0.72, 0.85] by bias-corrected and accelerated method; 5,000 resamples], as the confidence interval included zero. Thus, the hypothesis that self-efficacy mediates the effect of HIIT on negative affect was not supported in this exploratory analysis.

**Table 5:** Exploratory bootstrap mediation analysis (5,000 resamples; *N* = 55).

Path	B	SE	t	p	95% CI
a: Group → post self-efficacy	1.057	0.233	4.55	<0.001	[0.601, 1.514]
b: Post self-efficacy → post negative affect	0.085	0.355	0.24	0.811	[−0.611, 0.782]
c: Total effect (group → post negative affect)	−3.674	0.572	−6.42	<0.001	[−4.795, −2.553]
c′: Direct effect	−3.764	0.689	−5.46	<0.001	[−5.114, −2.414]
Indirect effect a × b (bootstrap)	0.090	0.395	—	—	[−0.750, 0.818]

X = group (1 = HIIT, 0 = control); M = post-intervention self-efficacy; Y = post-intervention negative affect. Covariates in all equations: baseline self-efficacy, baseline negative affect, sex, and age. Confidence interval for the indirect effect is the bootstrap percentile interval; the bias-corrected and accelerated interval was [−0.722, 0.853].

**Figure 6:**
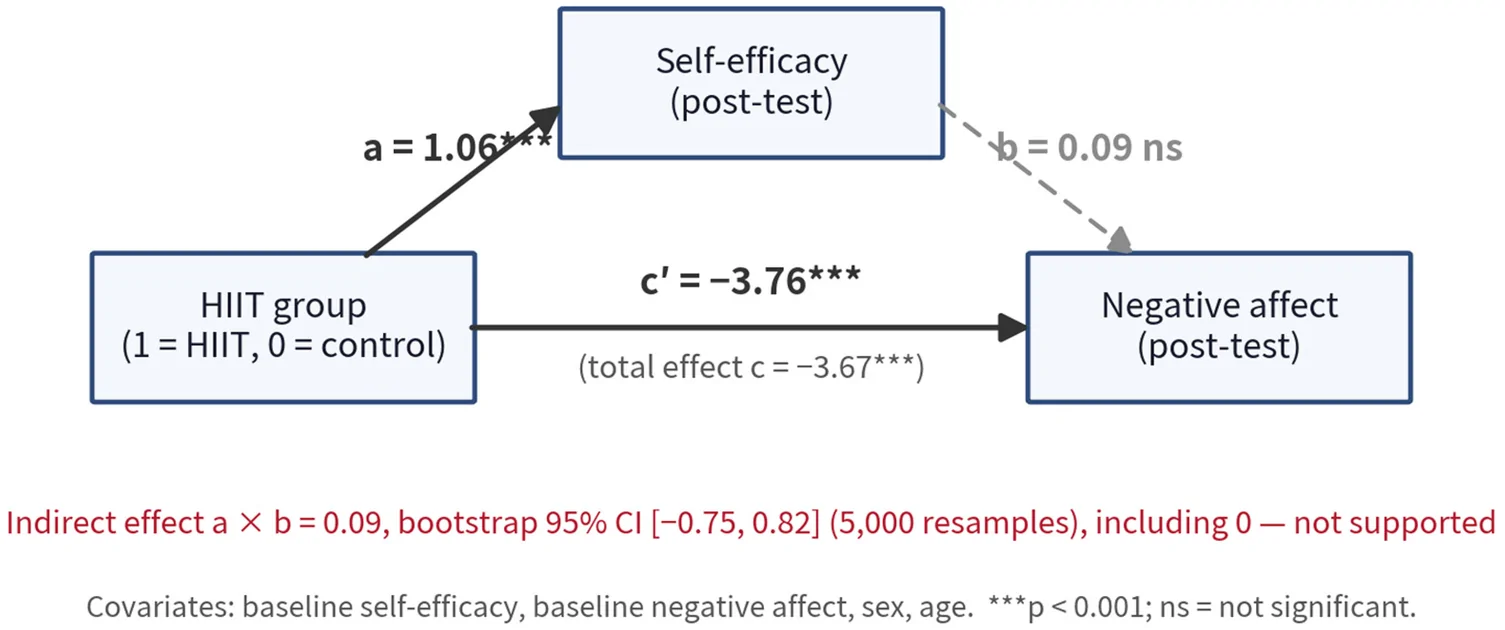
Exploratory mediation model with unstandardized coefficients. ***p < 0.001; ns, not significant. The bootstrap 95% confidence interval of the indirect effect included zero.

## 4 Discussion

In this school-based randomized controlled trial, 8 weeks of HIIT produced broad and significant benefits in adolescents with obesity. After adjustment for baseline values, the experimental group showed lower body weight, BMI, visceral and subcutaneous fat mass, body fat percentage, and fat mass, together with higher estimated VO₂max, lower negative affect, and higher self-efficacy than the control group; the effects on the primary psychological outcomes were large (partial *η*^2^ = 0.475 for negative affect and 0.290 for self-efficacy). Contrary to our initial hypothesis, the exploratory bootstrap mediation analysis did not support self-efficacy as a mediator of the effect of HIIT on negative affect once baseline values, sex, and age were taken into account.

### 4.1 Effects on body composition and cardiorespiratory fitness

The improvements in body composition observed here are consistent with previous randomized trials of school-based HIIT in adolescents with obesity (21, 31) and with meta-analytic evidence that HIIT is at least as effective as moderate-intensity continuous training for improving body composition (20). The pattern of results—reductions in fat-related measures with no significant loss of fat-free or muscle mass—suggests that the weight loss induced by the intervention was predominantly fat loss, which is the desirable profile for this population.

The cardiorespiratory findings require careful interpretation. Because BMI is a term in the equation used to estimate VO₂max, and BMI decreased in the experimental group, the nominal improvement in estimated VO₂max (adjusted difference 1.54 mL/kg/min) partly reflects this mathematical coupling. When baseline BMI was held constant, the effect was attenuated by roughly two-thirds (0.50 mL/kg/min) but remained significant, and the BMI-free lap count confirmed a genuine improvement in shuttle-run performance. We therefore interpret the findings as evidence of improved running performance and a probable, more modest improvement in aerobic fitness, and we recommend that future studies using prediction equations for VO₂max report similar sensitivity analyses.

### 4.2 Effects on negative affect

The reduction in negative affect in the experimental group is consistent with meta-analytic evidence that HIIT improves depressive symptoms compared with inactive controls (32), with large-scale network meta-analyses demonstrating that exercise alleviates depressive symptoms in children, adolescents, and young adults (33, 34), with reviews focusing specifically on youth likewise concluding that HIIT holds promise for improving mental health in this age group (35), and with broader evidence that physical activity protects against anxiety and negative emotional states (15, 36). By contrast, a 2024 meta-analysis of randomized trials in healthy individuals found no significant effect of HIIT on depressive and anxiety symptoms (37); together with our findings, this suggests that the emotional benefits of HIIT may be more pronounced in at-risk populations such as adolescents with obesity than in healthy samples, and that the existing evidence base remains heterogeneous. Several mechanisms may plausibly contribute, although none was directly assessed in the present study: HIIT has been shown to promote the release of endogenous opioid peptides such as endorphins and to modulate monoamine neurotransmitters (38, 39), and exercise training can improve hypothalamic–pituitary–adrenal axis function (36). At the experiential level, the alternating high- and low-intensity structure of HIIT reduces the monotony of continuous exercise, and its time-efficient, clearly defined tasks may be particularly suitable for adolescents with obesity, who often have a history of unsuccessful exercise attempts; affective and enjoyment responses to HIIT in this population have been reported to be comparable to those for moderate-intensity continuous training (40–42). Notably, psychological improvement was not statistically explained by the measured psychological mediator, echoing findings from multidisciplinary weight-management interventions in which psychological improvements were largely uncorrelated with objective physiological changes (43).

### 4.3 Effects on self-efficacy

HIIT significantly improved self-efficacy in adolescents with obesity, consistent with previous findings (44, 50, 51). According to Bandura's theory (45, 46), the four sources of self-efficacy were all plausibly engaged by the intervention. First, repeated completion of prescribed high-intensity bouts provided direct mastery experiences—the most powerful source of efficacy beliefs—for adolescents who had accumulated experiences of failure in physical activity. Second, observing peers with similar body sizes complete the same training provided vicarious experiences. Third, real-time encouragement and affirmation from instructors constituted verbal persuasion. Fourth, participants' interpretation of their physiological arousal during training may have been reframed from a threat signal into a sign of capability as fitness improved. The visible improvements in body composition and running performance over the 8 weeks likely reinforced these beliefs further.

### 4.4 Why was the mediating effect of self-efficacy not supported?

Although HIIT improved both negative affect and self-efficacy, the indirect effect of HIIT on negative affect through self-efficacy was not statistically supported after adjustment for baseline self-efficacy, baseline negative affect, sex, and age (bootstrap 95% CI [−0.75, 0.82]). This contrasts with cross-sectional studies in general student samples, in which self-efficacy has been reported to mediate the association between physical exercise and negative affect (16–18, 49). Several explanations should be considered. Moreover, given the modest sample size, this non-significant finding should not be interpreted as evidence of the absence of a mediating effect: the analysis may have been underpowered to detect a small indirect effect.

First, and most fundamentally, the mediator and the outcome were measured concurrently at the post-intervention assessment. Mediation is a causal, temporally ordered claim, and concurrent measurement of mediator and outcome cannot establish temporal ordering; reverse causation—that reduced negative affect enhanced self-efficacy—cannot be excluded. Methodological standards for mediation analysis recommend designs in which the mediator is measured before the outcome (29, 30, 47, 48); our two-wave design satisfies this requirement only partially, because baseline values were used as covariates rather than as temporal anchors for change. Second, the apparently significant b path in unadjusted analyses was largely driven by the cross-sectional correlation between self-efficacy and negative affect at the post-intervention assessment and by a modest baseline imbalance in self-efficacy (24.74 vs. 22.96, *p* = 0.102); once these were controlled, the b coefficient approached zero (B = 0.09, *p* = 0.811). This illustrates why baseline adjustment is essential in mediation analyses of trial data (30). Third, the direct effect of HIIT on negative affect remained large and significant (c′ = −3.76), suggesting that the emotional benefits of HIIT in this population may operate predominantly through pathways other than general self-efficacy—for example, neurobiological mechanisms (36, 38, 39), acute affective responses and enjoyment during exercise (40–42), or social processes in the group-based training context (peer support, instructor feedback, and shared mastery). These candidate mechanisms remain hypotheses to be tested with appropriately timed measurements. Finally, a non-specific factor deserves consideration. The experimental group received a structured and closely supervised intervention, whereas the control group attended regular physical education classes; differences in instructor attention, encouragement, peer interaction, and supervision may themselves have contributed to the improvements in self-efficacy and negative affect. The psychological benefits observed here should therefore be interpreted as effects of the HIIT program as a whole, including its social and supervisory context, rather than as effects of the exercise stimulus alone. Future trials could address this issue by including an attention-matched active control condition.

### 4.5 Limitations and future directions

This study has several limitations. First, the trial was not prospectively registered; although the outcomes and measurement procedures were prespecified in the ethics-approved protocol and all measured outcomes have been reported, selective reporting cannot be entirely ruled out. Second, and most important for the mediation question, self-efficacy and negative affect were measured at the same post-intervention time point, so temporal ordering cannot be established and the mediation analysis is exploratory; future studies should use at least three measurement waves (e.g., baseline, mid-intervention, and post-intervention) to separate the mediator from the outcome in time. Third, participants were drawn from a single school and the sample size was modest; subgroup effects by sex, degree of obesity, and baseline psychological status could not be examined; no *a priori* sample-size calculation was performed, and the mediation analysis in particular may have been underpowered to detect a small indirect effect. Fourth, the intervention lasted 8 weeks rather than the more common 12 weeks because of the school's academic schedule, and the durability of the effects is unknown. Fifth, dietary intake and out-of-school physical activity were not objectively monitored, so contributions of unmeasured lifestyle changes cannot be excluded. Sixth, blinding of participants and intervention staff was not feasible, although outcome assessors were blinded. Seventh, VO₂max was estimated from a prediction equation rather than measured directly, and its estimate is mathematically coupled to BMI; we addressed this with sensitivity analyses, but directly measured VO₂peak would be preferable. Eighth, maturation status (e.g., Tanner stage or age at peak height velocity) was not assessed. Ninth, multiple outcomes were tested without formal multiplicity adjustment; however, the primary outcomes (negative affect and self-efficacy) were prespecified, and their effects were large. Finally, only one HIIT protocol (15 s/15 s) was tested; future studies could compare different work-to-rest ratios and exercise modalities and could incorporate neuroendocrine indicators (e.g., cortisol, brain-derived neurotrophic factor, endorphins), electroencephalography, or functional near-infrared spectroscopy to clarify the mechanisms through which HIIT improves emotion. In addition, exercise intensity was monitored by post-bout radial pulse checks and RPE rather than by continuous heart-rate monitoring, which may not fully capture the peak intensity achieved during the 15-s intervals. Finally, the control condition was not matched to the intervention for instructor attention, encouragement, and supervision, so non-specific attention effects on the psychological outcomes cannot be excluded.

## 5 Conclusions

Eight weeks of school-based HIIT significantly improved body composition and running performance, reduced negative affect, and enhanced self-efficacy in adolescents with obesity, with large effects on the psychological outcomes. The exploratory mediation analysis did not support the hypothesis that self-efficacy mediates the effect of HIIT on negative affect after adjustment for baseline values; because the mediator and outcome were measured concurrently, this question remains open and should be addressed in longitudinal studies with temporally separated measurements. HIIT is a feasible, safe, and time-efficient intervention that can be implemented in school settings to improve both the physical and the emotional health of adolescents with obesity.

## Data Availability

The original contributions presented in the study are included in the article/Supplementary Material, further inquiries can be directed to the corresponding author
